# Preclinical reserpine models recapitulating motor and non-motor features of Parkinson’s disease: Roles of epigenetic upregulation of alpha-synuclein and autophagy impairment

**DOI:** 10.3389/fphar.2022.944376

**Published:** 2022-10-12

**Authors:** Yang Li, Qiao Yin, Bing Wang, Tingting Shen, Weifeng Luo, Tong Liu

**Affiliations:** ^1^ Department of Neurology and Clinical Research Center of Neurological Disease, The Second Affiliated Hospital of Soochow University, Suzhou, China; ^2^ Department of Neurology, Huzhou Central Hospital, Affiliated Central Hospital Huzhou University, Huzhou, China; ^3^ Institute of Pain Medicine and Special Environmental Medicine, Nantong University, Nantong, China

**Keywords:** Parkinson’s disease, non-motor symptoms, reserpine, methylation, autophagy

## Abstract

Reserpine is an effective drug for the clinical treatment of hypertension. It also induces Parkinson’s disease (PD)-like symptoms in humans and animals possible through the inhibition of monoamine vesicular transporters, thus decreasing the levels of monoamine neurotransmitters in the brain. However, the precise mechanisms remain unclear. Herein, we aimed to develop a preclinical reserpine model recapitulating the non-motor and motor symptoms of PD and investigate the underlying potential cellular mechanisms. Incubation of reserpine induced apoptosis, led to the accumulation of intracellular reactive oxygen species (ROS), lowered DNA methylation of alpha-synuclein gene, resulted in alpha-synuclein protein deposition, and elevated the ratio of LC3-II/LC3-Ⅰ and p62 in cultured SH-SY5Y cells. Feeding reserpine dose-dependently shortened the lifespan and caused impairment of motor functions in male and female Drosophila. Moreover, long-term oral administration of reserpine led to multiple motor and non-motor symptoms, including constipation, pain hypersensitivity, olfactory impairment, and depression-like behaviors in mice. The mechanistic studies showed that chronic reserpine exposure caused hypomethylation of the alpha-synuclein gene and up-regulated its expression and elevated the ratio of LC3-II/LC3-Ⅰ and expression of p62 in the substantia nigra of mice. Thus, we established preclinical animal models using reserpine to recapitulate the motor and non-motor symptoms of PD. Chronic reserpine exposure epigenetically elevated the levels of alpha-synuclein expression possible by lowering the DNA methylation status and inducing autophagic impairment *in vitro* and *in vivo*.

## 1 Introduction

Parkinson’s disease (PD) is a common neurodegenerative disease affecting more than six million people worldwide. PD is one of the main reasons for disability globally, and its incidence is increasing at a rate faster than that of other neurological diseases (Loh et al., 2021). The prevalence of PD increases with age, reaching its peak between 85 and 89 years old. Among PD patients, the incidence is higher in men compared with women (Bae et al., 2014). Although both motor and non-motor symptoms of PD have been identified, its current clinical treatment is oriented primarily toward the former (Wang M. et al., 2020b). The motor symptoms of PD include bradykinesia, muscular rigidity, resting tremors, and postural instability (Lim et al., 2018), while the non-motor symptoms include depression and anxiety, pain, autonomic dysfunction (e.g. constipation), cognitive disorders, and sleep disturbances (Krashia et al., 2019). Both motor and non-motor symptoms of PD seriously affect the quality of life of these patients and their caregivers, additionally leading to heavy social burden (Leão et al., 2015; Lim et al., 2018). Clinically, a multi-faceted approach is necessary for PD management, including medications [e.g. levodopa, (L-DOPA)], non-pharmacological therapy, and surgery (e.g. deep brain stimulation) (Vanegas-Arroyave et al., 2016; Pahwa et al., 2017). However, prevention and therapy for PD remain challenging.

Appropriate animal models of PD are crucial for studying the disease pathophysiology and developing new therapeutic strategies. Neurotoxin-based and/or genetic approaches are often utilized to develop PD models using rodents, zebrafish, and *drosophila* (Chia et al., 2020). Several neurotoxins are frequently used to establish PD models, including 6-hydroxydopamine (6-OHDA), 1-methyl-4-phenyl-1,2,3,6-tetrahydropyridine (MPTP), rotenone, and paraquat (Chia et al., 2020). Notably, the reserpine model is also effective for studying PD pathophysiology for a long period and screening anti-PD drugs. Carlsson et al. (1957), Carlsson and Carlsson (1989) first reported the close relationship between reserpine and PD, wherein they observed alleviation of reserpine-induced dyskinesia in rodents upon L-DOPA administration. It is considered that reserpine binds to the presynaptic vesicular monoamine-transporter 2 (VMAT2), leading to the blockade of vesicular uptake and synaptic release of the monoamine neurotransmitters, including dopamine, 5-hydroxytryptamine, and norepinephrine (Rijntjes and Meyer, 2019). The utility of the reserpine model has been confirmed for studying the roles of the monoamine system in the pathogenesis of PD (Leão et al., 2015). In addition, reserpine also induces cognitive and memory impairment, anxiety- and depression-like behaviors, sleep disturbance, and pain sensitization, in addition to typical dyskinesia (Skalisz et al., 2002; Bisong et al., 2010; Arora and Chopra, 2013; Santos et al., 2013; Liu et al., 2014). Thus, the reserpine model may be excellent for recapitulating motor and/or non-motor symptoms in PD (Leão et al., 2015). However, the contribution of alpha-synuclein deposition and autophagy to reserpine-induced PD-like symptoms remains elusive, which may be not directly linked to monoamine depletion activity of reserpine.

PD is a complex disease involving both genetic and environmental factors. Alpha-synuclein (encoded by *SNCA*) accumulation plays a critical role in the pathogenesis of PD (Pediaditakis et al., 2021). The expression of *SNCA* is essential for the development of PD, including gene duplications, triplications, and mutations (Valek et al., 2019). DNA methylation is an important epigenetic mechanism underlying the dysregulation of alpha-synuclein expression owing to the changes in gene dosages due to gene transcriptional regulation. For alpha-synuclein gene, CpG-1 is located in the first exon, while CpG-2 is in the first intron of *SNCA* (Miranda-Morales et al., 2017). Treatment with DNA methylation inhibitors decreases CpG-2 methylation and increases the mRNA and protein level expression of *SNCA* in SK-N-SH cells (Jowaed et al., 2010). DNA methylation levels are significantly lower in sorted post-mortem neuronal nuclei of the frontal cortex from PD specimens relative to that of normal controls (Gu et al., 2021). DNMT1 (DNA methyltransferase 1) expression is decreased significantly in cultured neuronal cells, post-mortem brain tissue from PD patients, and *SNCA* transgenic mice (Desplats et al., 2011).

Autophagy and the ubiquitin-proteasome system are responsible for the degradation of alpha-synuclein, which was detected in autophagolysosomes and lysosomes (Lee et al., 2015). Accumulation of alpha-synuclein can result in autophagic impairment, contributing to the pathogenesis of PD (Lee et al., 2015). Many pathogenic events in PD are directly or indirectly associated with autophagy dysregulation, which has been previously demonstrated in the brain of different animal neurotoxic models of PD (Lu et al., 2020). Therefore, understanding the mechanisms of epigenetic regulation and autophagic impairment is important to study the deposition of alpha-synuclein that implicated in PD.

In this study, we aimed to develop preclinical animal models of reserpine-induced PD showing motor and non-motor symptoms, and to identify the molecular and cellular mechanisms underlying the effects of reserpine *in vivo* and *in vitro*. We tested the hypothesis that reserpine treatment may induce motor and non-motor symptoms of PD through autophagic flux dysfunction and regulation of the DNA methylation status of *SNCA,* leading to increased protein expression of alpha-synuclein.

## 2 Material and methods

### 2.1 Animals

Adult male ICR mice (6–8 weeks old and weighing 30 g) were obtained from the Shanghai SLAC Laboratory Animal Co., Ltd. (Shanghai, China). The environment of the rearing room was maintained at an ambient temperature of 22 ± 2°C; 40%–60% humidity, and a 12-h light and dark cycle (8 a.m.–8 p.m.). A total of 3-5 mice were housed per cage with free access to water and food. The Ddc-GAL4 fly lines were obtained from Bloomington Stock Center (Indiana University, Bloomington, IN, United States). Cornmeal-molasses medium was used for maintaining the strains at 25°C. All animal experimental procedures in this study were approved by the Animal Ethics Committee of Soochow University.

### 2.2 Cell culture and treatment

The human neuroblastoma SH-SY5Y cells were obtained from the American Type Culture Collection (ATCC, CRL-2266), which show moderate levels of dopamine-β-hydroxylase. The human neuroblastoma SH-SY5Y cells were grown in high-sucrose DMEM supplemented with 10% fetal bovine serum (FBS) in a humidified incubator with 5% CO_2_ at 37°C. Reserpine was dissolved in saline containing 10% DMSO at indicated concentrations (5 μM, 10 μM, 25 μM, 50 μM, and 100 µM); SH-SY5Y cells were incubated with reserpine.

### 2.3 CCK-8 assay

Briefly, 3000 cells/well were seeded in 96-well plates containing a complete growth medium; the CCK-8 kit was used (C6005, NCM Biotech, China), and the assay was conducted following the manufacturer’s instructions (Wang et al., 2018). Subsequently, 10 µl of the test drug was added to the cultured cells at indicated durations and these cells were incubated for 2 h at 37°C. The absorbance at 450 nm was measured using a microplate reader to assess the cell viability.

### 2.4 Annexin V-FITC/PI apoptosis assay

Briefly, cells were obtained after digestion with EDTA-free trypsin, washed with PBS, and resuspended in 100 μl of 1 × binding buffer (He et al., 2018). To these cells, 5 μl of Annexin V-FITC and 10 μl of PI staining solution were added and incubated in dark at room temperature for 10–15 min. Subsequently, 400 μl of 1 × binding buffer was added to the cells, following which these were incubated on ice, and examined by flow cytometry within an hour.

### 2.5 DNA methylation assay

Briefly, DNA was extracted from SH-SY5Y cells using the Genomic DNA Extraction Kit (QIAGEN) (Hong et al., 2016). Methylation was assayed and PCR amplification was performed using the Qiagen Epi Tech Bisulfite Kit (Qiagen, 59104) following the manufacturer’s instructions. Pyrosequencing Q96 and Q48 were used to generate data. Finally, the results of pyrophosphate sequencing were analyzed. Table 1 lists the primer sequences used in this study.

**TABLE 1:** List of primers.

Primer	5′ to 3′	5′ modification
*SNCA*-1F (207bp)	AGT​TGT​TGG​AGG​AGA​TAG​GTA	
*SNCA* -1R (Q96)	TCC​TTA​ATA​ATC​TCC​CTT​TCA​CC	5′-Biotin
*SNCA* -1S	TGGAGGAGATAGGTAG	
*SNCA* -2F (173bp)	GGA​AGT​GTA​AGG​AGG​TTA​AGT​TAA​T	
*SNCA* -2R (Q48)	ATC​CAC​CCC​CCC​CCT​CAA​CTA​TCT​A	5′-Biotin
*SNCA* -2S	AGG​TGG​TAA​AGG​GTT​AAT​AA	
*ATG3*-1F (104bp)	AGA​AGT​TTT​TTT​GAG​GGA​GGT​AA	
*ATG3*-1R (Q96)	AAT​CAC​ACA​TAC​CCA​ATA​AAA​CTC​TTC​ACT	5′-Biotin
*ATG3*-1S	TTTTTGAGGGAGGTAAT	
*ATG3*-2F (269bp)	TGG​AGA​AGT​TAT​AGT​AGG​TAT​AGG	5′-Biotin
*ATG3*-2R (Q96)	CCC​TTC​CTT​CTC​ACT​CTC​TTA	
*ATG3*-2S	CTTCTCACTCTCTTACC	

### 2.6 Measurement of intracellular reactive oxygen specie levels in SH-SY5Y cells

Briefly, following the manufacturer’s instructions, using 2,7-dichlorodihydrofluorescein diacetate and dimethyl sulfoxide as stock solutions, intracellular ROS levels were measured as described previously (Zhou et al., 2017). Before treatment, SH-SY5Y cells were seeded in 6-well plates for at least 12 h. Incubation with or without reserpine (100 μmol/L) was for 24 h followed by 3 washes with cold PBS after replacing the medium with DCFH-DA (25 mol/L). The fluorescence intensity was assessed using a Zeiss fluorescence confocal microscope LSM 800 (Oberkochen, Germany). The ZEN software was used to analyze the images. To perform quantitative flow cytometry (FC500; Beckman Coulter, Brea, CA) analysis, adherent cells were treated with reagents and fluorescent probes, followed by suspension in 500 µl PBS. Measurement and analysis of fluorescence intensity were performed with Cxp (FC500; Beckman Coulter).

### 2.7 Drugs

Reserpine injection was purchased from King York Pharmaceutical (Tianjin, China). All mice were randomly divided into control or reserpine groups. Pilot experiments showed the daily water (containing reserpine) intake per mouse in a single cage was about 6 ml. Reserpine was mixed daily into the drinking water of the mice at corresponding concentrations of 0.9, 3, or 9 μg/ml, thus the reserpine intake per mouse was about 0.18, 0.6, or 1.8 mg/kg/day. The dose converts to a human equivalent dose of 0.014, 0.048, 0.136 mg/kg (using conversion factor 12.3 for mouse to human according to FDA guidelines).

Water with or without reserpine in the bottle was freshly prepared every 3 days. L-DOPA and pramipexole were procured from Shandong Xinhua Pharmaceutical and Boehringer Ingelheim Pharma GmbH & Co. KG, respectively. Mice received intraperitoneal injections of 10 mg/kg of L-DOPA and pramipexole.

### 2.8 Battery of behavioral tests

#### 2.8.1 Rotarod test

The Rotarod system (ZH-300, Zhenghua Co. Ltd., China) was used to evaluate motor ability as described previously (Li Y. et al., 2019b). Before the test, all mice were subjected to rotarod training once a day for 3 consecutive days. First, the mice were kept on the rod without falling off for approximately 5 min. The initial speed of the rotarod was 0 rpm, which was accelerated to 20 rpm within 20 s. Only the mice that exercised on the rotarod for 5 min at a speed of 20 rpm qualified for subsequent analyses. Mice that fell off were excluded. After training, the rotarod test was conducted and the on-rod durations were recorded.

#### 2.8.2 Pole-climbing test

The pole (1 cm diameter and 60 cm in length) was used to test the climbing ability of mice (Frawley et al., 2020). The mice were placed on the thicker end of a pole to ensure that their heads were facing downwards, while their tails were facing upwards. The mice climbed to the end of the pole with their heads facing downwards. The movement time for reaching the end of the pole was recorded.

#### 2.8.3 Open-field test

The open field system (XR-XZ301, Shanghai Xin Ruan Information Technology Co. Ltd, Shanghai, China) was used to test the locomotion of mice as described previously (Li Y. et al., 2019b). The mice were placed in the experimental room for 30 min for acclimatization. Next, they were placed vertically in the bottom center of a box (40 cm × 40 cm × 40 cm) and filmed continuously for an observation period of 10 min. The animals were recorded in real-time using far-infrared sensors for evaluated the moved distance, the number of times the animal crossed the center, and their tracks.

#### 2.8.4 Forced swimming test

Briefly, mice were placed in a quiet experimental room for 30 min to acclimatize (Li Y. et al., 2019b). A glass beaker was filled with approximately 2.8 L of water (24°C–26°C). Before the test, the mice were placed in the water-filled glass beaker for 15 min. Subsequently, they were dried using a dryer and placed back in their original cages. On the day of the test, the mice were placed in the water-filled glass beaker for 6 min. Their immobility times were recorded in the last 4 min for statistical analysis.

#### 2.8.5 Tail suspension test

The experiment was conducted in a quiet room (Li C. L. et al., 2019). Mice were fixed to the suspension stand, 50 cm above the floor, using medical tape approximately 1 cm from their tail tips. The observation period lasted for 6 min after the mice were fixed. The immobility time in the last 4 min was recorded for statistical analysis.

#### 2.8.6 Sucrose solution preference test

Mice were singly housed in cages for 24 h (Li Y. et al., 2019). After acclimatization, the following steps were performed: first 24-h period, two bottles of 1% sucrose solution were placed in each cage; in the second 24-h period, one of the bottles was replaced with water without sucrose and the positions of the two bottles were swapped after 12 h; third 24-h period, the mice did not have libitum access to food or water, and the fourth 24-h period-the new bottle was weighed (one each of daily drinking water and 1% sucrose solution). The positions of the two bottles were swapped after 12 h and their weights were recorded. Finally, the weights of the two bottles were recorded after 24 h. The following calculations were performed: Sucrose solution preference index (%) = sucrose solution consumption/(sucrose solution consumption + pure water consumption) x 100%.

#### 2.8.7 Burying food pellet test

Mice had access to water but not food for 24 h (Wang H. et al., 2020a). 3-cm-thick bedding was laid flat in a 20 cm × 25 cm x 40 cm in the new feeding cage, wherein the feed was buried. Mice were randomly placed and the time from placing them in the cage till they found the food and held it in their orbit was recorded.

#### 2.8.8 Mechanical pain threshold

The mice were placed in a test box for 1 h for acclimatization. When the mice were quiet, their pain thresholds were tested using Von Frey filaments (6g, 8g, 15g, 26g, 60g, 100g, 180g, and 300g) (Chen et al., 2021). A positive response (if foot withdrawal behavior was present) was recorded as 1, else as 0. During the experiment, each stimulus was provided 10 times on either side. The positivity rate was calculated and recorded as the statistical value.

#### 2.8.9 Defecation test

Each week, before and after reserpine administration, the animals were immediately transferred to clean, empty cages for 24 h and fed as described above (Hu et al., 2019). The feces were collected from the cage, counted, and recorded as a statistical value. Additionally, the feces were photographed for assessing their morphology.

### 2.9 Real-time quantitative PCR analysis

Briefly, RNA was extracted using the TRIzol method (Invitrogen, 15596026) (Li Y. et al., 2019b). Total RNA was extracted from the SH-SY5Y cells and the substantia nigra of mice brains treated with or without reserpine. Subsequently, reverse transcription and amplification were performed using the K1622, Revert Aid First Strand cDNA kit (Thermo Fisher Scientific, Waltham, United States). The primer sequences used in this study were as follows: For SH-SY5Y cells, *DNMT1* (FW, AGA​ACG​GTG​CTC​ATG​CTT​ACA; RV, CTC​TAC​GGG​CTT​CAC​TTC​TTG), *DNMT3a* (FW, AGT​ACG​ACG​ACG​ACG​GCT​A; RV, CAC​ACT​CCA​CGC​AAA​AGC​AC), *DNMT3b* (FW, AGG​GAA​GAC​TCG​ATC​CTC​GTC; RV, GTG​TGT​AGC​TTA​GCA​GAC​TGG), *MBD2* (FW, GCA​AGC​CTC​AGT​TGG​CAA​G; RV, ATC​GTT​TCG​CAG​TCT​CTG​TTT), *MBD4* (FW, CAG​GAA​CAG​AAT​GCC​GTA​AGT; RV, CCT​TGT​GGG​CTG​ATA​AAG​TAC​AC), *TDG* (FW, TGA​AGC​TCC​TAA​TAT​GGC​AGT​TG; RV, TTC​CAC​TGG​TTG​TTT​TGG​TTC​T), *GADD45a* (FW, CCC​TGA​TCC​AGG​CGT​TTT​G; RV, GAT​CCA​TGT​AGC​GAC​TTT​CCC), *GAPDH*(FW, CAG​GAG​GCA​TTG​CTG​ATG​AT; RV, GAA​GGC​TGG​GGC​TCA​TTT). For mice, *Dnmt1*(FW, AAG​AAT​GGT​GTT​GTC​TAC​CGA​C; RV, CAT​CCA​GGT​TGC​TCC​CCT​TG), *Dnmt3a* (FW, GGC​CGA​ATT​GTG​TCT​TGG​TG; RV, CCA​TCT​CCG​AAC​CAC​ATG​AC), *Dnmt3b* (FW, AGC​GGG​TAT​GAG​GAG​TGC​AT; RV, GGG​AGC​ATC​CTT​CGT​GTC​TG), *Mbd2* (FW, AGA​ACA​AGG​GTA​AAC​CAG​ACC​T; RV, ACT​TCA​CCT​TAT​TGC​TCG​GGT), *Mbd4* (FW, GGA​CAA​CAG​AGT​CCG​TGG​AG; RV, ATC​ACC​AGG​TCC​TTT​CCA​TCT), *Tdg* (FW, AAG​TTC​CTA​ACA​TGG​CAG​TCA​C; RV, ATT​TCT​TCG​ACG​TAG​CAG​GTT​T), *Gadd45a* (FW, CCG​AAA​GGA​TGG​ACA​CGG​TG; RV, TTA​TCG​GGG​TCT​ACG​TTG​AGC), *Gapdh* (FW, TGT​GAA​CGG​ATT​TGG​CCG​TA; RV, GGC​CTC​ACC​CCA​TTT​GAT​GT). The SYBR Green Master Mix (Bi make, B21703) was used. The CT values were recorded on the ABI 7500 real-time fluorescent PCR instrument (ABI 7500, Life technology, United States). The results were calculated using the 2 ^ ^(−∆∆CT)^ method.

### 2.10 Western blotting

SH-SY5Y cells and the substantia nigra from mice brains treated with or without reserpine were analyzed. Western blotting was conducted as reported previously. RIPA lysates containing protease and phosphatase inhibitors were used to obtain the protein samples (Li Y. et al., 2019b). Protein concentrations were determined using the BCA quantification method (Pierce, Rockford, IL). The same quantity of protein samples (30 μg) was added per well in the 10% and 15% sodium dodecyl-sulfate polyacrylamide gel electrophoresis (SDS-PAGE). Following electrophoresis proteins were transferred to the 0.45 μm PVDF membranes. The blots were blocked in 5% milk for 1 h. Blots were incubated overnight at 4°C with primary antibodies against alpha-synuclein (Abcam, ab212184), LC3 (Abcam, ab51520), p62 (CST, 8025), DNMT3b (Abcam, ab119282) and GAPDH (Mesgen biotechnology, MAN1002). On the next day, the blots were incubated with the secondary antibody, (goat anti-rabbit IgG (H + L) HRP (Mesgen, MAN4001), or goat anti-mouse IgG (H + L) HRP (Mesgen, MAN4002)) for 1 h at room temperature. The ECL detection solution was prepared as follows: A: B = 1:1, mixed homogeneously. Blots were imaged for detecting luminescence. Finally, the grayscale values were analyzed using Fiji Image J software.

### 2.11 Detection of dopamine levels in brain specimens by high-performance liquid chromatography

Striatum and substantia nigra of mice brains treated with or without reserpine were analyzed. Briefly, at the 12th week after reserpine treatment, animals were anesthetized using isoflurane and intracardially perfused with PBS (Cao et al., 2016). Dissected brains were weighed and homogenized in 400 ml 0.4 mol/L perchloric acid using an ultrasonic homogenizer (Microsonic, Dortmund, Germany). The samples were centrifuged at 15000 rpm for 10 min at 4°C, filtered through the 0.22 μm syringe filter, and stored at −80°C. Measurements of dopamine concentrations were performed by reverse-phase HPLC and electrochemical detection (SHIMADZU, LC-6A, Japan). For this reverse phase column, 0.1 mol/L NaAc was injected with 10% methanol (pH 5.1) and 0.1 mol/L EDTA-Na2. The flow rate was 1 ml/min. Finally, a standard curve was plotted based on the area under the peak of the standard solution and the samples were analyzed.

### 2.12 Immunohistochemistry

Substantia nigra of mice brains treated with or without reserpine were analyzed. Briefly, mice were anesthetized using isoflurane and perfused with 4% paraformaldehyde in 0.01 M phosphate buffer (pH 7.4) (Wang et al., 2018). The whole brain was dissected; fixed using 4% paraformaldehyde overnight, and cryoprotected in 30% sucrose solution for 48 h before coronal sections were cut using a microtome (Leica SM 2010R, Leica). PBS and PBST (0.25% Triton X-100 in PBS) solutions were used for washes following which sections were blocked for 1 h in PBST containing 5% goat serum. The sections were incubated in the primary antibody (TH, rabbit, CST, 58844S) overnight at 4°C followed by incubation with a fluorescently labeled secondary antibody (goat anti-rabbit, Alexa Fluor 488, Abcam, ab150081) at room temperature for 1h. Sections were mounted using Fluoromount-G (0100-01, Southern Biotech). Using the Carl Zeiss LSM 800 laser-scanning confocal microscope (Oberkochen, Germany), the images were captured.

### 2.13 Survival curve and climbing assay in flies

Twenty newly enclosed male or female flies were collected in a plastic tube (15 cm in length and 1.5 cm in diameter) containing food (Wang et al., 2018). The flies were transferred into tubes with fresh food every alternate day. The survival of flies was recorded every day. Based on the survival curve, 50% survival time was used to compare the survival ratio among different groups. We performed at least 3 independent experiments.

To analyze the locomotor ability of flies, a negative geotaxis assay was performed. Briefly, 20–22 flies from each group were assayed for climbing ability weekly in a vertical plastic tube (15 cm in length and 1.5 cm in diameter). After allowing the flies to habituate for 30 min at room temperature and gently rapping to the bottom of the tube, the number of flies that could climb to the test line within 10 s was counted. The half-pass time or the time at which 50% of the flies were able to climb above the test line was used to compare the climbing abilities among different groups. We performed 3 independent experiments.

### 2.14 Data analyses

Statistical values were analyzed using the GraphPad Prism 8.3 software. All values were expressed as mean ± SEM. The unpaired Student’s t-test was used for comparing the differences between the two groups. For comparisons involving more than two groups, one-way ANOVA was performed and Dunnett’s post hoc test was used for comparing the mean against that of the control group while Tukey’s post hoc test was used for comparison among means of different groups. Two-way ANOVA with Tukey’s post hoc test was performed for comparing all means to that of the control. *p* < 0.05 indicated a statistically significant difference.

## 3 Results

### 3.1 Reserpine promotes apoptosis in cultured SH-SY5Y cells

**FIGURE 1:**
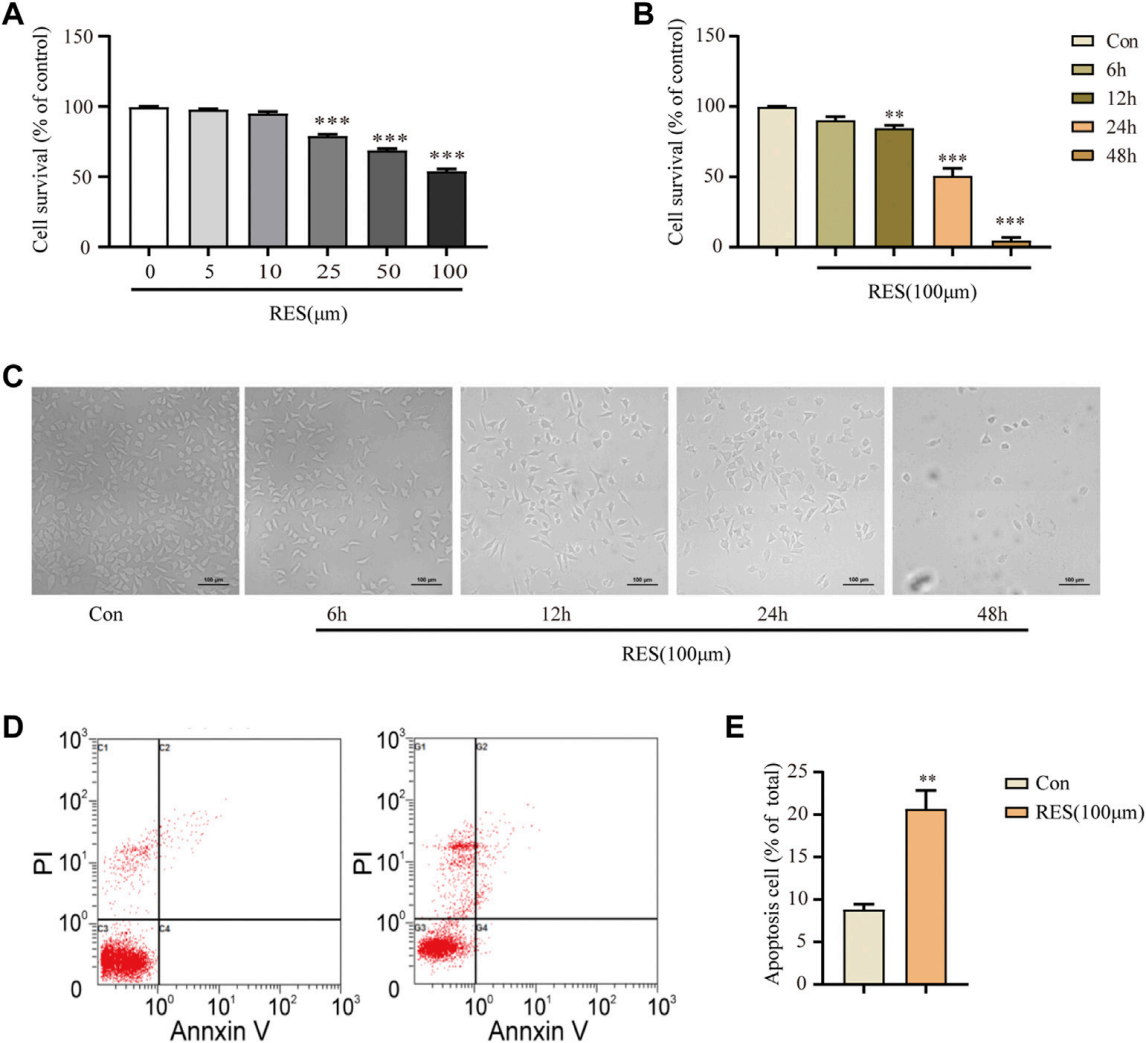
Reserpine treatment in SH–SY5Y cells induced apoptosis. **(A)** The cell survival in SH–SY5Y cells decreased with increasing concentration of reserpine by CCK8 assay (*n* = 4 for each group, one-way ANOVA with Dunnett post hoc test, ***p < 0.001 vs. control). **(B,C)** The cell survival decreased with time in SH–SY5Y cells treated with 100 μM reserpine by CCK8 assay (*n* = 4 for each group, one-way ANOVA with Dunnett post hoc test, **p < 0.01, ***p < 0.001 vs. control). Representative images are shown in **(C)**. **(D,E)** Flow cytometry showed that apoptosis rate increased in SH–SY5Y cells treated with 100 μM reserpine for 24 h (*n* = 4 for each group, student t-test, **p < 0.01 vs. control). All data are expressed as mean ± SEM; Con, control; RES, reserpine; h, hour.

A CCK-8 assay was performed to evaluate the effects of different concentrations of reserpine (0, 5, 10, 25, 50, and 100 μM) on neuronal cell survival after treatment for 24 h. The cell survival rate decreased in a dose-dependent manner. In the SH-SY5Y cells treated with reserpine (100 μM) for 24 h, it was 53.81% (one-way ANOVA, F _(5, 18)_ = 212.0, *p* < 0.0001, Figure 1A). Thus, 100 μM of reserpine was determined as the optimal dose for further experiments. To assess the time-dependent effects of reserpine on the SH-SY5Y cell survival, these cells were treated with reserpine (100 μM) for indicated time points (6, 12, 24, and 48 h). The cell survival rate decreased in a time-dependent manner in 12, 24, and 48-h treatment groups (one-way ANOVA, F _(4, 15)_ = 176.8, *p* < 0.0001, Figures 1B,C). The apoptosis rate of SH-SY5Y cells following 24-h reserpine treatment (100 μM) was measured using the Annexin V-PI kit. The apoptosis rate of SH-SY5Y cells significantly increased in reserpine-treated cells as compared to the control (Student’s *t*-test, t = 5.241, *p* = 0.0019, Figures 1D,E). The above results suggested that reserpine treatment induced robust apoptosis in the cultured SH-SY5Y cells.

### 3.2 Reserpine induces autophagy impairment in SH-SY5Y cells

**FIGURE 2:**
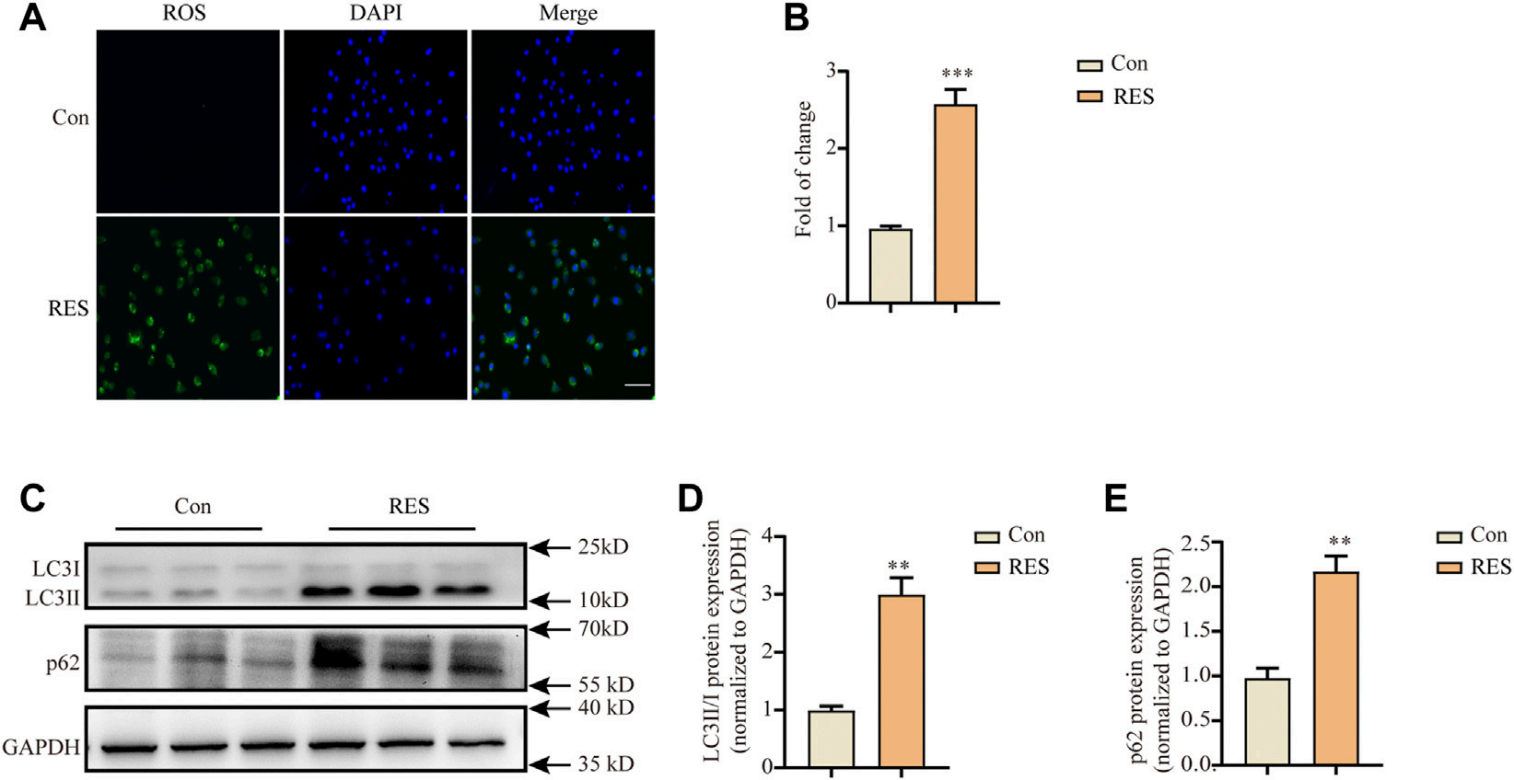
Reserpine treatment in SH-SY5Y cells induced the accumulation of intracellular ROS and autophagic dysfunction. **(A,B)** Representative fluorescence images for intracellular ROS stained with DCFH-DA probe showed that reserpine induced significant accumulation of intracellular ROS, which was suppressed by the antioxidants, NAC and PBN; scale bar: 100 μm. **(A,B)** are representative and statistical plots, respectively (*n* = 3 for each group, student t-test, *p < 0.05, ***p < 0.001 vs. control). **(C)** Representative western blotting images for LC3 and P62 expression. **(D)** The ratio of LC3-II/LC3-I protein expression increased (*n* = 3 for each group, student t-test, **p < 0.01 vs. control). **(E)** The levels of p62 protein expression increased (*n* = 3 for each group, student t-test, **p < 0.01 vs. control). All data are expressed as mean ± SEM; Con, control; RES, reserpine; NAC, *N*-acetyl-L-cysteine; PBN, *N*-tert-butyl-α-phenylnitrone.

Oxidative stress occurs in reserpine-induced rodent models of PD (Fernandes et al., 2012; Santos et al., 2013). Proteins affected by oxidative stress are cleared mainly through the autophagy-lysosomal pathway (Lama et al., 2021). Therefore, we measured intracellular ROS levels after reserpine treatment. Incubation with reserpine (100 μM) for 24 h significantly increased the intracellular ROS levels in SH-SY5Y cells (Student’s *t*-test, t = 8.648, *p* < 0.001, Figures 2A,B).

Autophagy dysfunction critically contributes to the pathogenesis of PD (Lama et al., 2021). Therefore, we investigated whether reserpine treatment led to autophagy dysfunction *in vitro*. Western blotting was performed after treating SH-SY5Y cells with reserpine (100 μM) for 24 h. Reserpine (100 μM) treatment significantly increased the ratio of LC3-II/LC3-Ⅰ protein expression (Student’s *t*-test, t = 7.068, *p* = 0.0021; Figures 2C,D), and the protein levels of p62 were also enhanced (Student’s *t*-test, t = 6.008, *p* = 0.0039; Figures 2C,E). Thus, these results suggested that reserpine treatment in SH-SY5Y cells might cause autophagic impairment.

### 3.3 Reserpine significantly changes the DNA methylation levels of SNCA gene in SH-SY5Y cells

**FIGURE 3:**
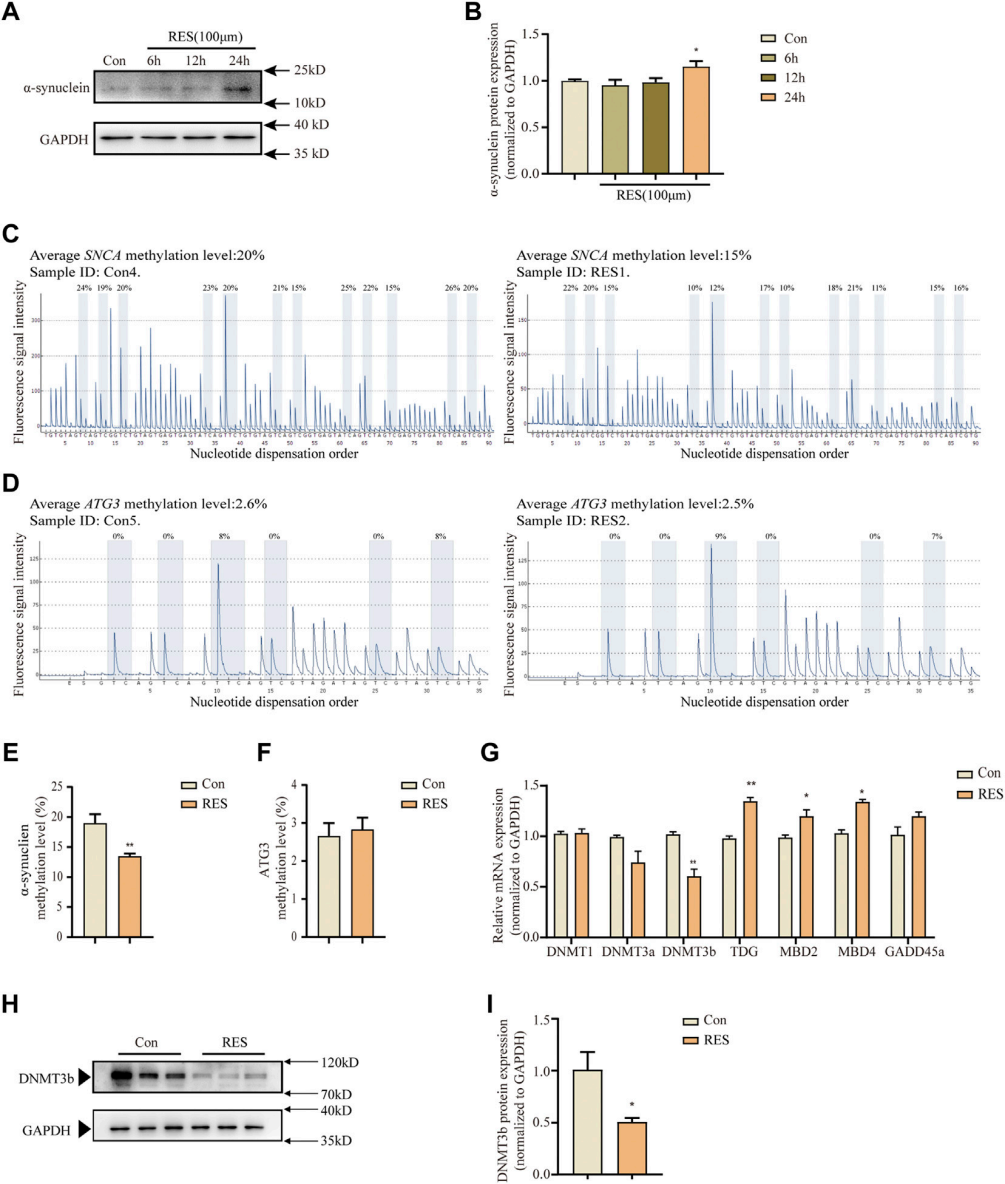
Reserpine treatment in SH–SY5Y cells induced decreased DNA methylation levels. **(A,B)** Reserpine treatment resulted in elevated protein expression of SNCA in SH–SY5Y cells (n = 3 for each group, one-way ANOVA with Dunnett post hoc test, *p < 0.05 vs. control). **(C,E)** Reserpine treatment decreased DNA methylation of *SNCA* for a subset of CpG by pyrosequencing analysis. **(C,E)** are representative samples and statistical analysis, respectively (*n* = 6 for each group, student t-test, **p < 0.01 vs. control). **(D,F)** Reserpine treatment did not change the DNA methylation levels of *ATG3*. **(D,F)** are representative samples and statistical analysis, respectively **(G)** Differential expression analysis for methylation-related enzymes by qRT-PCR (*n* = 5 for each group, student t-test, *p < 0.05, **p < 0.01 vs. control). **(H,I)** Reserpine treatment resulted in decreased protein expression of DNMT3b in SH-SY5Y cells (*n* = 3 for each group, student t-test, * *p < 0.05 vs. control). All data are expressed as mean ± SEM; Con, control; RES, reserpine.

Importantly, alpha-synuclein aggregation and autophagic impairment are closely related and contribute to the pathogenesis of PD (Bento et al., 2016; Di Maio et al., 2018). To examine the changes in the expression of alpha-synuclein in SH-SY5Y cells following reserpine treatment, western blotting was performed to assess protein levels of alpha-synuclein at different time points (6 h, 12 h, and 24 h) following reserpine (100 μM) treatment. The levels of alpha-synuclein expression significantly increased following reserpine treatment for 24 h (one-way ANOVA, F _(3, 8)_ = 4.517, *p* = 0.0392, Figures 3A,B).

Epigenetic regulatory mechanisms underlying PD, including histone modifications and DNA methylation status of *SNCA* (Sancandi et al., 2020) have received increasing attention. Moreover, ATG3 plays a crucial role in the conversion of LC3 from type I to type II. Therefore, we detected the alterations in DNA methylation levels of *SNCA* and *ATG3* following reserpine treatment by pyrophosphate sequencing for the CpG islands in these genes. DNA methylation levels of *SNCA* (Student’s *t*-test, t = 3.614, *p* = 0.0047, Figures 3C,E), but not for *ATG3* (Figures 3D,F) were significantly lowered. Furthermore, the qRT-PCR analysis showed alterations in the expression of DNA methylation-related enzymes, including *DNMT1*, *DNMT3a*, *DNMT3b*, *TDG*, *MBD2*, *MBD4*, and *GADD45a* following reserpine treatment. Among these genes, the levels of *DNMT3b* expression significantly decreased (Student’s *t*-test, t = 6.919, *p* = 0.0023, Figure 3G); however, those of *TDG* (Student’s *t*-test, t = 7.369, *p* = 0.0018, Figure 3G), *MBD2* (Student’s *t*-test, t = 4.182, *p* = 0.0139, Figure 3G), and *MBD4* (Student’s *t*-test, t = 3.791, *p* = 0.0193, Figure 3G) were significantly elevated. In previous study, 6-OHDA and MPP^+^ induce down-regulation of DNMT3b and DNMT3a (Yang et al., 2017). However, in this study, the mRNA levels of DNMT3b were downregulated. So, we also tested the protein levels of DNMT3b by western blot. The levels of DNMT3b expression significantly decreased following reserpine treatment for 24 h (Student’s *t*-test, t = 2.88, *p* = 0.045, Figures 3H,I). Thus, these results suggested that reserpine treatment resulted in lowered DNA methylation of *SNCA* in SH-SY5Y cells, which may have resulted in the overexpression of alpha-synuclein.

### 3.4 Reserpine treatment induces motor impairment in Ddc-GAL4 flies

**FIGURE 4:**
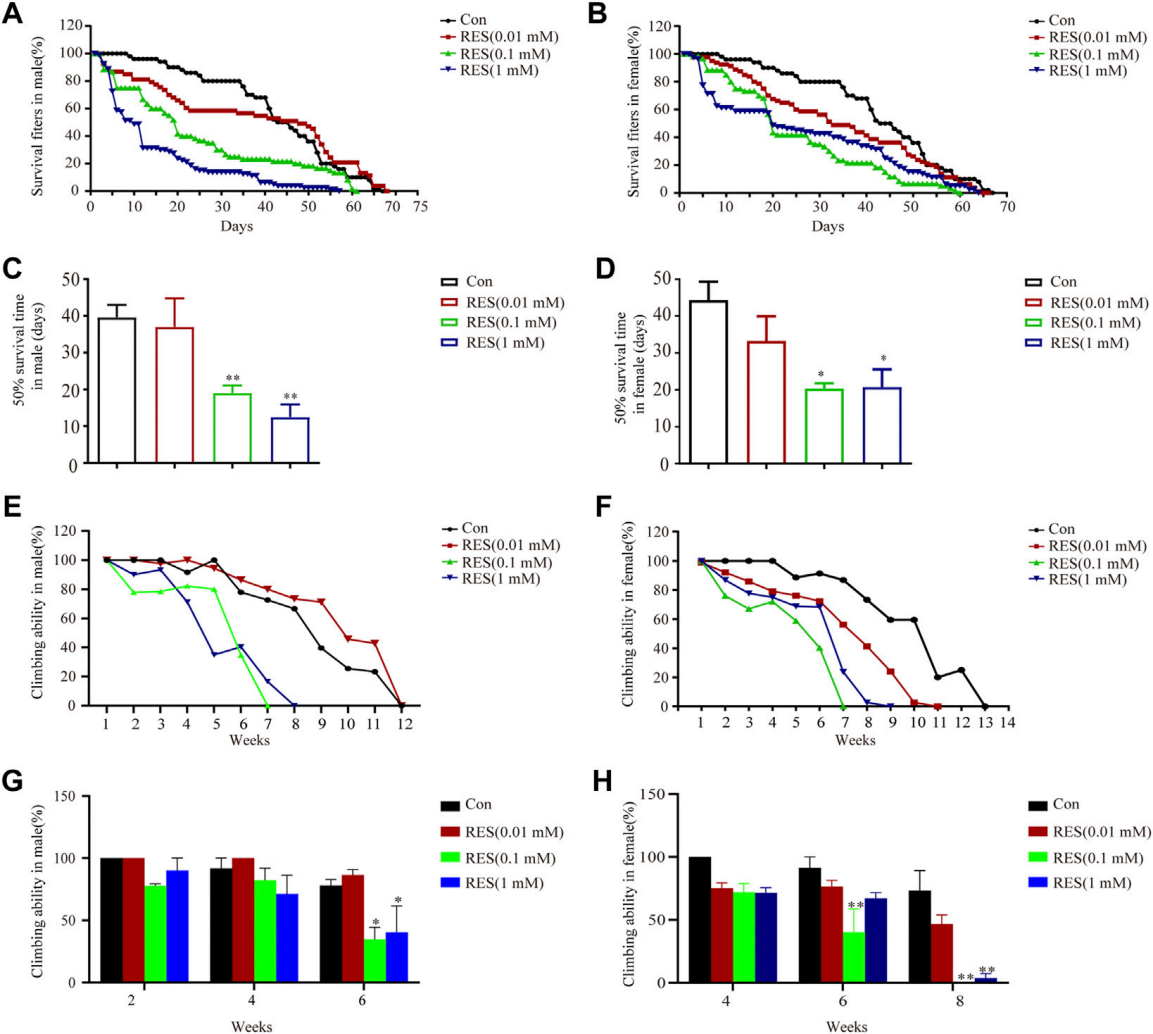
Reserpine treatment decreased survival and impaired climbing ability in flies. **(A,B)** Reserpine treatment decreased the lifespan of males and females, respectively, as compared to control flies. **(C,D)** Reserpine significantly shortened the 50% survival time in male and female flies (*n* = 3 for each group, one-way ANOVA with Tukey’s post hoc test, **p < 0.01 vs. control and *p < 0.05 vs. control, respectively). **(E,F)** Reserpine resulted in a significant decrease in climbing ability in male and female flies. **(G,H)** The effectiveness of reserpine was dose-dependent in male and female flies (*n* = 3 for each group, two-way ANOVA with Tukey’s post hoc test, *p < 0.05 vs. control and **p < 0.01 vs. control, respectively). All data are presented as mean ± SEM; Con, control; RES, reserpine.

After we demonstrated that reserpine treatment caused epigenetic regulation of expression of alpha-synuclein, autophagy impairment, and apoptosis in SH-SY5Y cells, we aimed to determine whether reserpine cause PD-related symptoms *in vivo*. When flies were fed on reserpine, their lifespan reduced significantly. Reserpine significantly shortened the 50% survival time in both male (one-way ANOVA, F _(3, 11)_ = 10.33, *p* = 0.0016, Figures 4A,C) and female (one-way ANOVA, F _(3, 10)_ = 4.356, *p* = 0.0331, Figures 4B,D) Ddc-GAL4 flies. Flies were placed gently at the bottom of a vial in order to measure the climbing ability. The climbing ability of male and female Ddc-GAL4 flies was impaired after reserpine treatment relative to the controls (Figures 4E,F). The climbing ability was attenuated in a dose-dependent manner in both males and females (two-way ANOVA, F _Dose__(3, 24)_ = 8.122, *p* = 0.0007; F _Time__(2, 24)_ = 13.08, *p* = 0.0001; F _Interaction__(6, 24)_ = 1.228, *p* = 0.3270; Figure 4G; two-way ANOVA, F _Dose__(3, 24)_ = 21.10, *p* < 0.0001; F _Time__(2, 24)_ = 36.74, *p* < 0.0001; F _Interaction__(6, 24)_ = 2.782, *p* = 0.0338; Figure 4H). Thus, these results suggested feeding flies with reserpine caused PD-like symptoms, including shortened lifespan and impaired climbing ability.

### 3.5 Oral administration of reserpine induces motor symptoms of Parkinson’s disease in mice

**FIGURE 5:**
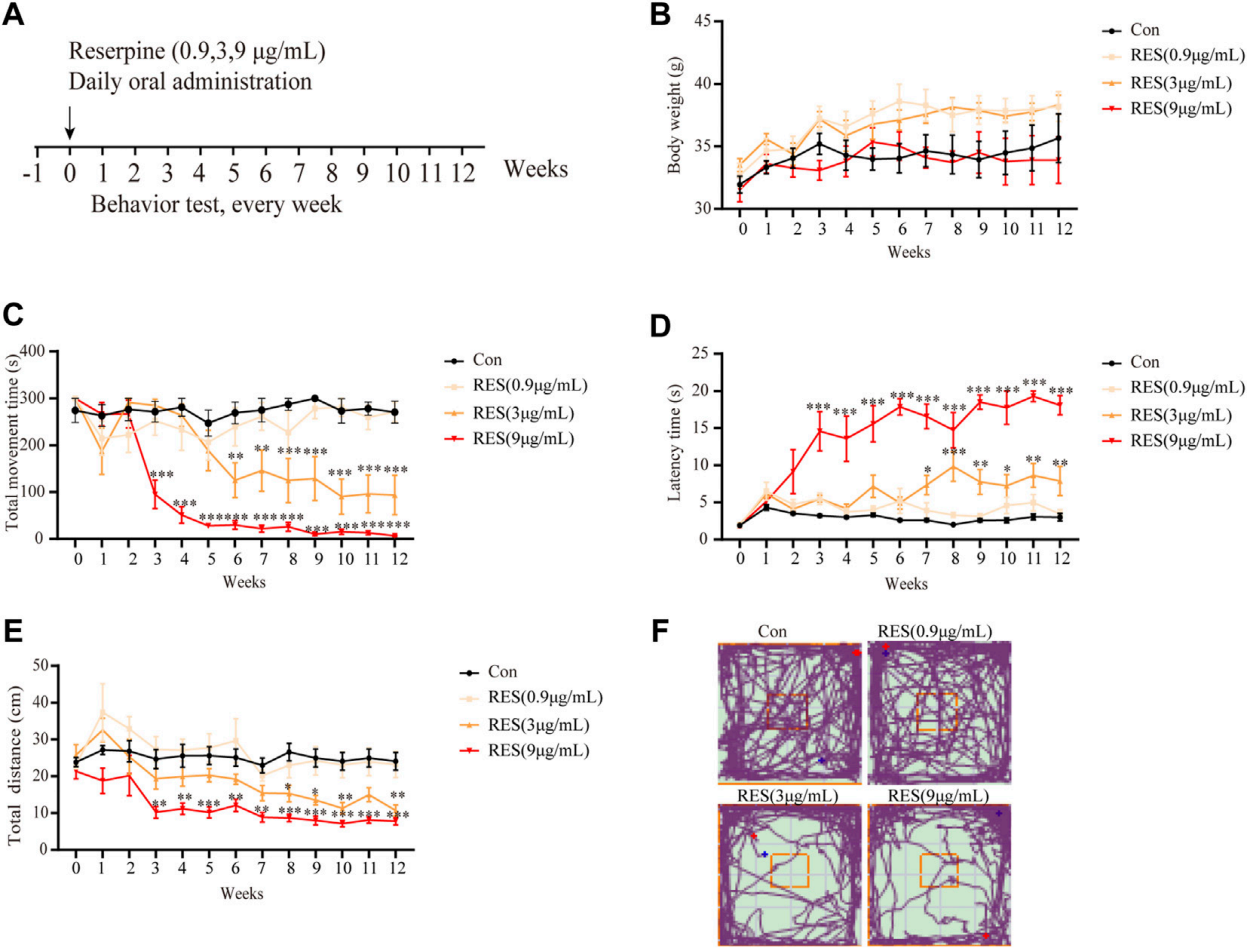
Reserpine induced PD-like motor symptoms in mice. **(A)** Flow chart of the experimental design. **(B)** Changes in body weights of mice induced by different doses of reserpine (*n* = 7 for each group, two-way ANOVA with Tukey’s post hoc test). **(C)** The movement time of mice decreased upon induction with different doses of reserpine in the rotarod test (*n* = 7 for each group, two-way ANOVA with Tukey’s post hoc test, **p < 0.01, ***p < 0.001 vs. control). **(D)** The latency time of mice increased upon induction with different doses of reserpine in the pole-climbing test (*n* = 7 for each group, two-way ANOVA with Tukey’s post hoc test, *p < 0.05, **p < 0.01, ***p < 0.001 vs. control). **(E)** Moved distances reduced in the open-field test upon induction with different doses of reserpine (*n* = 7 for each group, two-way ANOVA with Tukey’s post hoc test, *p < 0.05, **p < 0.01, ***p < 0.001 vs. control). **(F)** Representative plots for the movement trajectory. All data are presented as mean ± SEM; Con, control; RES, reserpine.

A previous study demonstrated that a single intraperitoneal (i.p.) injection of reserpine caused monoamine depletion in the brain, leading to severe but transient motor symptoms of PD in mice (Colpaert, 1987). We modified this method by oral administration of different concentrations of reserpine in the drinking water for 12 weeks to establish a preclinical PD mouse model (Figure 5A).

Reserpine (0.9, 3, and 9 μg/ml) in the drinking water was administered to male mice for 12 weeks. A battery of behavioral tests was performed to determine whether reserpine caused motor symptoms of PD. No significant changes in body weights were observed between the reserpine-treated and the control mice (Figure 5B). No significant differences in the motor functions of the mice between the lowest dose of reserpine (0.9 μg/ml)-treated and control groups were observed. Dyskinesia, a symptom of PD, was observed in 3 μg/ml and 9 μg/ml reserpine-treated groups. In the rotarod test, the total movement time significantly decreased in both 3 μg/ml and 9 μg/ml reserpine-treated groups (two-way ANOVA, F _Dose (12,312)_ = 9.932, *p* < 0.0001; F _Time__(3,312)_ = 118.1, *p* < 0.0001; F _Interaction__(36,312)_ = 5.176, *p* < 0.0001; Figure 5C). In the pole-climbing test, the latency time was significantly prolonged in the 3 μg/ml and 9 μg/ml reserpine-treated groups (two-way ANOVA, F _Dose (12,455)_ = 9.674, *p* < 0.0001; F _Time (3,455)_ = 195.2, *p* < 0.0001; F _Interaction__(36,455)_ = 4.751, *p* < 0.0001; Figure 5D). In the open field test, the total movement distance showed a significant reduction in both 3 μg/ml and 9 μg/ml reserpine-treated groups (two-way ANOVA, F _Dose (12,312)_ = 7.376, *p* < 0.0001; F _Time (3,312)_ = 71.69, *p* < 0.0001; F _Interaction (36,312)_ = 1.143, *p* = 0.2700; Figure 5E). A representative graph of the track at week 12 is shown in Figure 5F. Taken together, these results indicated that oral administration of reserpine induced motor symptoms of PD in mice.

### 3.6 Oral administration of reserpine induces non-motor symptoms of Parkinson’s disease in mice

**FIGURE 6:**
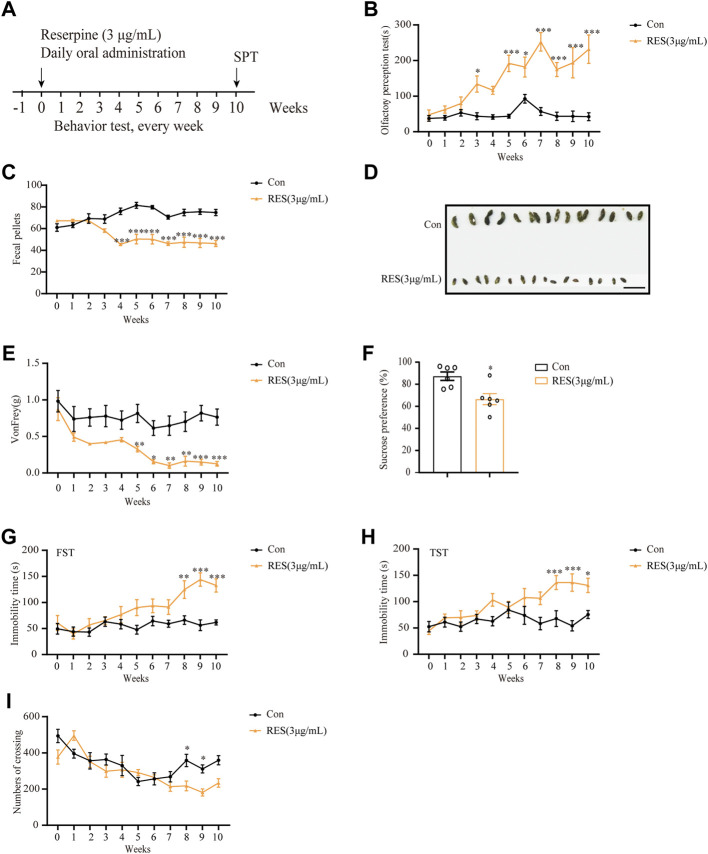
Reserpine induced PD-like non-motor symptoms in mice. **(A)** Flow chart of the experiment. **(B)** Reserpine (3 μg/ml) increased the time for olfactory perception in mice (*n* = 6 for each group, two-way ANOVA with Sidak post hoc test, *p < 0.05, **p < 0.01, ***p < 0.001 vs. control). **(C)** Reserpine (3 μg/ml) decreased fecal pellets (*n* = 10 for each group, two-way ANOVA with Sidak post hoc test, ***p < 0.001 vs. control). **(D)** Reserpine (3 μg/ml) induced changes in the shape of the stool; scale bar: 1 cm. **(E)** Reserpine (3 μg/ml) decreases mechanical pain threshold by von Frey test (*n* = 11 for each group, two-way ANOVA with Sidak post hoc test, *p < 0.05, **p < 0.01, ***p < 0.001 vs. control). **(F)** The sucrose preference rate decreased in mice administered with reserpine (3 μg/ml) (*n* = 6 for each group, student t-test, *p < 0.05 vs. control). **(G)** The immobility time in the forced swimming test increased in mice administered with reserpine (3 μg/ml) (*n* = 10 for each group, two-way ANOVA with Sidak post hoc test, **p < 0.01, ***p < 0.001 vs. control). **(H)** The immobility time in the tail suspension test increased in mice administered with reserpine (3 μg/ml) (*n* = 9 for each group, two-way ANOVA with Sidak post hoc test, *p < 0.05, ***p < 0.001 vs. control). **(I)** The number of crossing events in the central area decreased in mice administered with reserpine (3 μg/ml) (*n* = 9 for each group, two-way ANOVA with Sidak post hoc test, *p < 0.05 vs. control). All data are presented as mean ± SEM; Con, control; RES, reserpine.

Subsequently, 3 μg/ml reserpine was chosen as the dose for further investigations. The experimental design of various behavioral tests is shown in Figure 6A. Mice treated with reserpine (3 μg/ml) exhibited significant non-motor symptoms of PD relative to the control group. From the third week, the olfactory perception of mice in the reserpine group significantly reduced as compared to the control group (two-way ANOVA, F _Drug (10, 198)_ = 6.103, *p* < 0.0001; F _Time__(1, 198)_ = 47.48, *p* < 0.0001; F _Interaction (10, 110)_ = 5.898, *p* < 0.0001; Figure 6B). From the fourth week, reserpine-treated mice developed robust constipation, as reflected by a significant decline in the number of fecal pellets as compared to the control group (two-way ANOVA, F _Drug (10, 198)_ = 1.952, *p* = 0.0404; F _Time (1, 198)_ = 211.4, *p* < 0.0001; F _Interaction__(10, 198)_ = 12.42, *p* < 0.0001; Figure 6C), and the short and dry appearance (Figure 6D). From the fifth week, the mechanical pain thresholds of mice in the von Frey test were found to significantly decrease in reserpine-treated as compared to the control group (two-way ANOVA, F _Drug__(10, 220)_ = 4.672, *p* < 0.0001; F _Time__(1, 220)_ = 97.52, *p* < 0.0001; F _Interaction__(10, 220)_ = 1.491, *p* = 0.1491; Figure 6E). As for depression-like behaviors, the immobility time in both forced swimming and tail suspension tests was significantly prolonged in reserpine-treated mice as compared to the control from the 8th week (two-way ANOVA, F _Drug (10, 198)_ = 6.080, *p* < 0.0001; F _Time (1, 198)_ = 47.47, *p* < 0.0001; F _Interaction (10, 198)_ = 3.426, *p* = 0.0004, Figure 6G; two-way ANOVA, F _Drug (10, 176)_ = 4.345, *p* < 0.0001; F _Time (1, 176)_ = 41.01, *p* < 0.0001; F _Interaction (10, 176)_ = 2.984, *p* = 0.0017, Figure 6H). At the 10th week, the sucrose preference rate reduced significantly in reserpine-treated mice as compared to the control (Student’s *t*-test, t = 3.294, *p* = 0.0081, Figure 6F). For anxiety, at the eighth week, the number of crossing events in central area in the open field test decreased significantly in the reserpine-treated as compared to the control group (two-way ANOVA, F _Drug (10, 176)_ = 10.17, *p* < 0.0001; F _Time (1, 176)_ = 11.74, *p* = 0.0008; F _Interaction (10, 176)_ = 3.223, *p* = 0.0008, Figure 6I). Thus, oral administration of reserpine induced several non-motor symptoms of PD in mice.

### 3.7 Effects of different drugs on motor and non-motor symptoms in reserpine-treated mice

**FIGURE 7:**
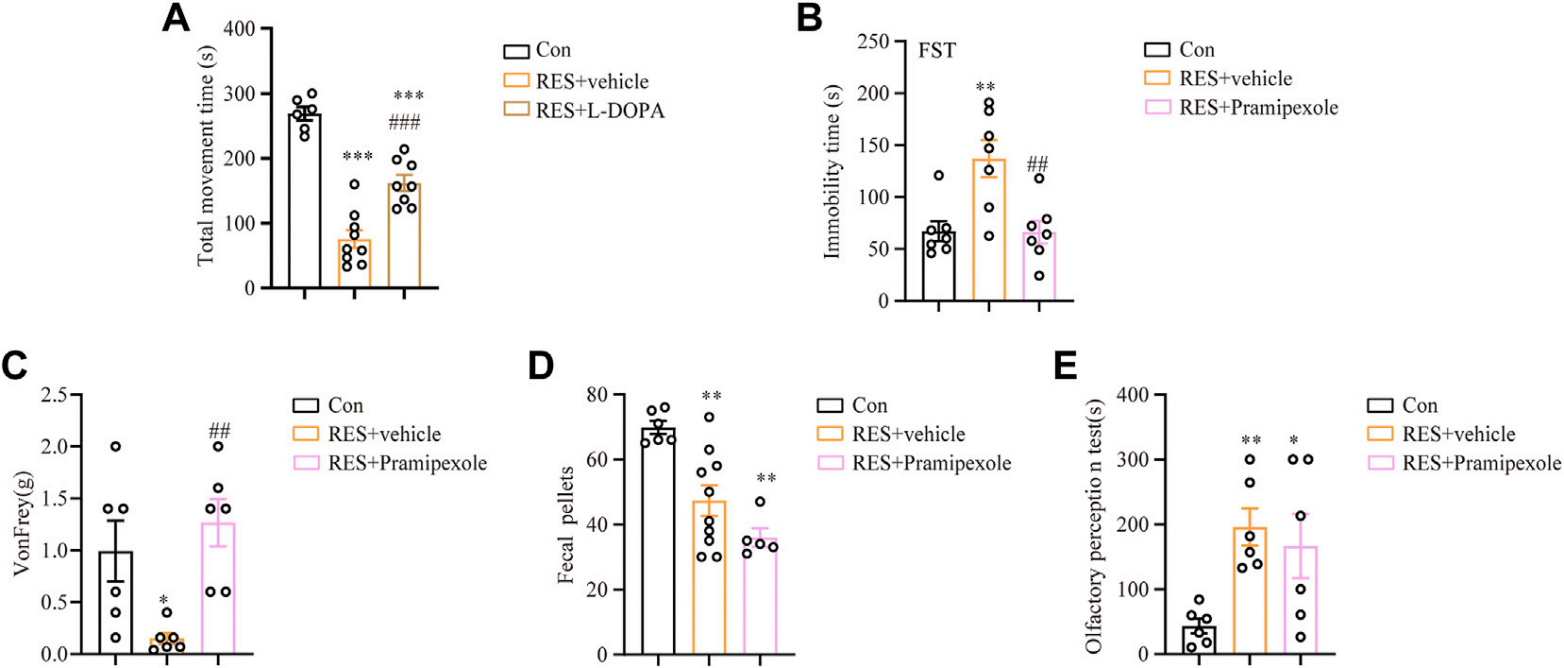
Effects of L-DOPA and pramipexole against reserpine-induced PD-like symptoms. **(A)** L-DOPA increased the movement time against reserpine-induced reduction in mice (n = 9 for each group, one-way ANOVA with Tukey’s post hoc test, ***p < 0.001 vs. control; ###*p* < 0.001 vs. RES). **(B)** The immobility time in the forced swimming test decreased upon pramipexole treatment in reserpine-induced mice (*n* = 7 for each group, one-way ANOVA with Tukey’s post hoc test, **p < 0.01 vs. control; ##*p* < 0.01 vs. RES). **(C)** Pramipexole upgraded the mechanical pain threshold in reserpine-induced mice (*n* = 6 for each group, one-way ANOVA with Tukey’s post hoc test, *p < 0.05 vs. control; ##*p* < 0.01 vs. RES). **(D)** Pramipexole was not effectively decreased the number of fecal pellets (*n* = 6 for each group, one-way ANOVA with Tukey’s post hoc test, **p < 0.01 vs. control). **(E)** Pramipexole was ineffective in reducing the olfactory perception time (*n* = 6 for each group, one-way ANOVA with Tukey’s post hoc test, *p < 0.05, **p < 0.01 vs. control). All data are presented as mean ± SEM; Con, control; RES, reserpine.

To test the validity of the reserpine mouse model, different clinically common used drugs were chosen for the treatment of reserpine-induced PD symptoms in mice. First, L-DOPA is the conventional drug used to manage PD symptoms (Zhou and Tan, 2020). Continuous injections of L-DOPA significantly relieved reserpine-induced motor symptoms in mice, as evidenced by increased movement time in the rotarod test (one-way ANOVA, F _(2, 20)_ = 53.14, *p* < 0.0001, Figure 7A). Second, we also tested pramipexole (Frampton, 2014) in reserpine mouse model. In the forced swimming test, the immobility time of the reserpine-treated mice significantly decreased following pramipexole treatment (one-way ANOVA, F _(2, 18)_ = 9.276, *p* = 0.0017, Figure 7B), suggesting pramipexole relieves PD-related depression symptoms. In the von Frey test, the mechanical pain threshold increased substantially following pramipexole treatment in the reserpine-treated group (one-way ANOVA, F _(2, 15)_ = 7.249, *p* = 0.0063, Figure 7C), indicating pramipexole alleviates PD-induced pain hypersensitivity. However, the number of fecal pellets remained unchanged after pramipexole treatment (Figure 7D). Moreover, olfactory impairment was also not affected by pramipexole treatment (Figure 7E). Together, these results suggested that pramipexole may be useful for alleviating multiple non-motor symptoms, including depression and pain, but not for constipation and olfactory impairment.

### 3.8 Reserpine induces autophagy impairment and low methylation levels in the substantia nigra of mouse

**FIGURE 8:**
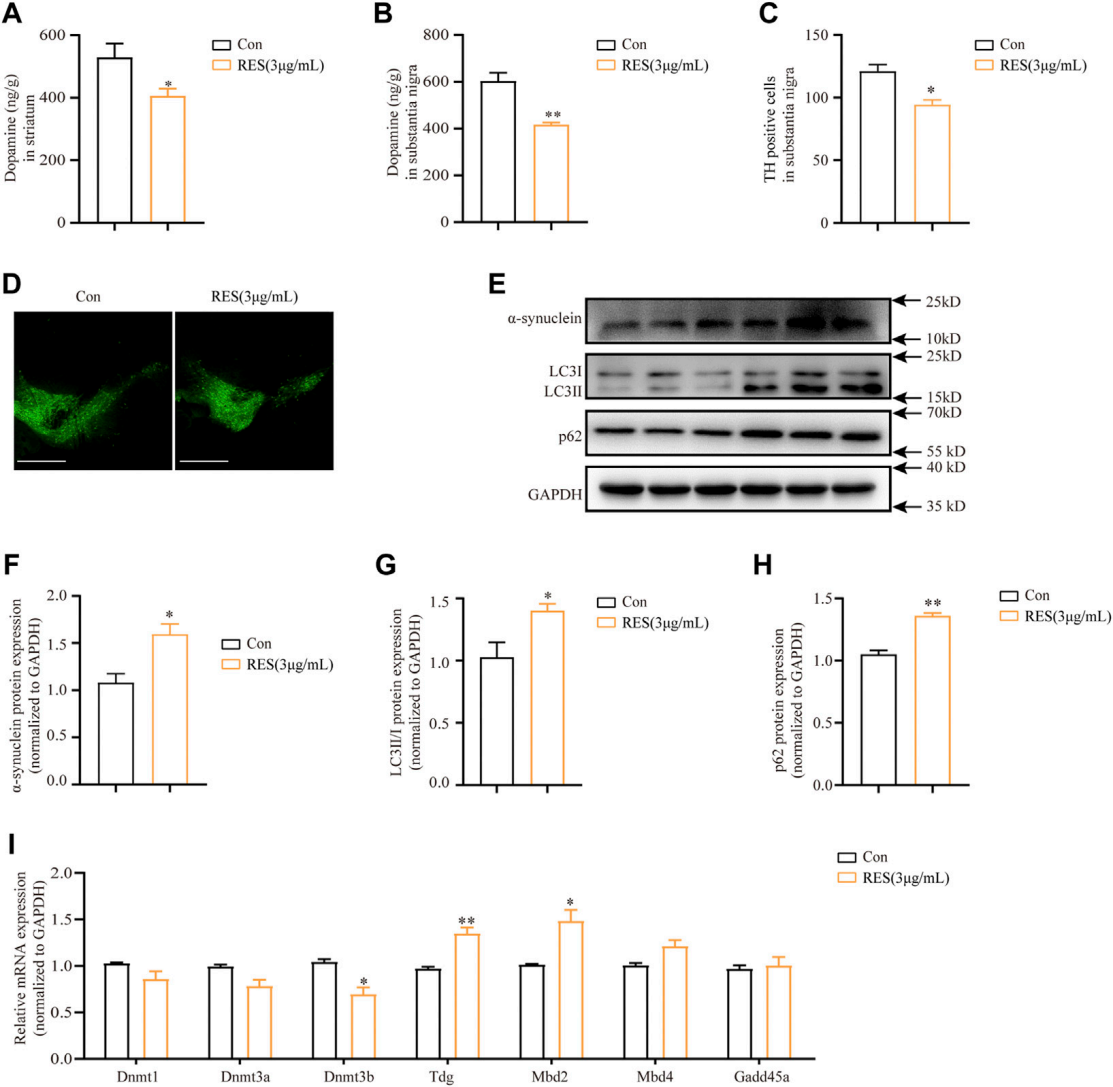
Reserpine induced autophagic dysfunction and lowers DNA methylation levels in mice. **(A)** The protein levels of dopamine were assayed by HPLC in the striatum (*n* = 3 for each group, student t-test, *p < 0.05 vs. control). **(B)** The protein levels of dopamine were assayed by HPLC in the substantia nigra (*n* = 3 for each group, student t-test, **p < 0.01 vs. control). **(C,D)** TH fluorescence intensity decreased in substantia nigra; scale bar: 100 μm (*n* = 3 for each group, student t-test, *p < 0.05 vs. control). **(E)** Representative blots of SNCA, LC3, and p62 protein expression in the substantia nigra. **(F–H)** Are statistical plots (*n* = 3 for each group, student t-test, *p < 0.05 **p < 0.01 vs. control). **(I)** Changes in mRNA expression of methylation-related enzymes in the substantia nigra following reserpine treatment (*n* = 5 for each group, student t-test, *p < 0.05 **p < 0.01 vs. control). All data are presented as mean ± SEM; Con, control; RES, reserpine.

To examine the changes of dopamine levels in mice brains following reserpine treatment, the substantia nigra and striatum were subjected to an HPLC assay. HPLC analysis showed that the dopamine levels were significantly decreased in the striatum (Student’s *t*-test, t = 2.448, *p* = 0.0499, Figure 8A) and substantia nigra (Student’s *t*-test, t = 5.107, *p* = 0.0069, Figure 8B) in reserpine-treated mice compared with the controls. Immunohistochemistry showed that the proportion of tyrosine hydroxylase (TH)-positive cells reduced significantly in reserpine-treated mice, as compared to the control at the 10th week (Student’s *t*-test, t = 4.077, *p* = 0.0151, Figures 8C,D). Additionally, Western blotting was performed for the proteins extracted from the substantia nigra of mice. The protein expression levels of alpha-synuclein increased markedly in reserpine-treated mice as compared to the control (Student’s *t*-test, t = 3.644, *p* = 0.0030, Figures 8E,F). Western blotting analysis showed that reserpine treatment significantly elevated the ratio of LC3-II/LC3-Ⅰ protein expression (Student’s *t*-test, t = 2.890, *p* = 0.0446, Figures 8E,G). Additionally, the levels of p62 protein expression also significantly increased (Student’s *t*-test, t = 8.173, *p* = 0.0012, Figures 8 E,H). These findings suggested that reserpine treatment induced autophagic impairment. Further, qRT-PCR analysis showed that mRNA levels of *Dnmt3b* decreased significantly (Student’s *t*-test, t = 3.298, *p* = 0.0300, Figure 8I), while those of *Tdg* (Student’s *t*-test, t = 7.109, *p* = 0.0021, Figure 8I), while *Mbd2* (Student’s *t*-test, t = 3.888, *p* = 0.0177, Figure 8I) were significantly elevated in the reserpine-treated mice compared with the controls.

## 4 Discussion

Clinically, reserpine is used as an anti-hypertensive and anti-psychotic drug. However, it often causes PD-like symptoms in patients (Santos et al., 2013). Although reserpine can deplete monoamines in the brain, the mechanism underlying the effects of reserpine on PD-like symptoms remains unclear (Leão et al., 2015). In this study, we elucidated the molecular mechanism underlying the effects of reserpine on PD-like symptoms using cell and animal models. Our results demonstrated that reserpine reduced the viability of SH-SY5Y cells and induced autophagic impairment. Moreover, reserpine treatment increased alpha-synuclein protein expression, possibly by lowering DNA methylation of *SNCA* gene in SH-SY5Y cells. Feeding reserpine shortened lifespan and impaired climbing ability in flies. Long-term oral administration of reserpine induced motor and non-motor symptoms in mice, which recapitulated PD-like behaviors. Moreover, reserpine treatment increased the LC-II/LC-I ratio and p62 and altered the levels of DNA methylation-related enzymes in the substantia nigra of mice. Thus, our results suggested that reserpine caused PD-like symptoms, possibly through epigenetic upregulation of alpha-synuclein and autophagic impairment.

### 4.1 Reserpine-induced Parkinson’s disease animal models

Neurotoxin-induced PD animal models are important for studying mechanisms and screening new anti-PD drugs. Most PD animal models exhibit motor symptoms and nigrostriatal degeneration, with a few non-motor symptoms. At present, genetic models show less evidence of non-motor symptoms than drug-induced animal models (Lama et al., 2021). For example, in the 1-methyl-4-phenyl-1,2,3,6-tetrahydropyridine (MPTP) mouse model, motor symptoms related to PD, including incoordination, tremor, and bradykinesia have been reported (Li C. L. et al., 2019a). However, MPTP causes only a few symptoms that are experienced in the late stages, referred to the dopamine-related motor symptoms (Wakeman et al., 2017). The subacute MPTP mouse model shows anhedonia (non-motor symptom) following 7 days of treatment (Tang et al., 2020). In addition, 6-OHDA-treated animals exhibits motor symptoms (Bonito-Oliva et al., 2014), but non-motor symptoms induced by 6-OHDA remain elusive, especially for depression-like behaviors (Branchi et al., 2010). Although the injection routes of reserpine and the duration of its administration differ substantially in the literatures (Leão et al., 2015), previous studies demonstrate that reserpine can induce typical dyskinesia, cognitive and memory impairment, anxiety and depression-like behaviors, sleep disturbance, and nociceptive sensitization (Skalisz et al., 2002; Bisong et al., 2010; Arora and Chopra, 2013; Santos et al., 2013; Liu et al., 2014). In this study, owing to the possible roles of the gut-brain axis in PD (Lolekha et al., 2021), reserpine was orally administered in mice. We performed behavioral tests for motor and non-motor symptoms related to PD for a relatively long period in mice. Our results clearly showed that oral administration of reserpine induced motor and non-motor symptoms, including constipation, pain hypersensitivity, olfactory perception, and depression in mice. Moreover, reserpine-fed flies showed impaired climbing ability. Collectively, we have established reserpine-induced PD animal models, which displayed both motor and non-motor symptoms, which effectively recapitulate the clinical symptoms of PD.

Previous studies show that single reserpine administration induces depletion of all monoamines (such as dopamine, norepinephrine, and 5-hydroxytryptamine) in the brain, causing severe but transient dyskinesia in rodents (Colpaert, 1987; Bourin, 1990). In the present study, dopamine levels in the substantia nigra and striatum significantly reduced after reserpine treatment in mice. Previous reports showed after repeated injections of low-dose reserpine in middle-aged (6-7-month-old) rats and mice, TH expression levels and TH-positive cell numbers in the nigrostriatal markedly decrease (Santos et al., 2013; Silva-Martins et al., 2021). However, 30 days after the last reserpine injection, a partial recovery of striatal TH expression and TH-positive cell counts (Santos et al., 2013) has been observed. In the present study, oral administration of reserpine induced a decrease in levels of TH expression in the substantia nigra along with reduced cell viability of SH-SY5Y cells, suggesting that reserpine is able to damage the dopaminergic neurons. In addition, reserpine also induces alpha-synuclein deposition, along with a reduction of TH in the substantia nigra, which represents the major hallmark of the pathogenesis of PD.

Importantly, this reserpine model showed a good response to commonly used clinical drugs, such as L-DOPA and pramipexole. The most common treatment for PD is L-DOPA, a dopamine replacement agent (Gandhi and Saadabadi, 2022). Previous studies demonstrated that L-DOPA alleviates the motor symptoms induced by reserpine (Carlsson et al., 1957; Carlsson and Carlsson, 1989). In the present study, we also tested the possible therapeutic efficacy of L-DOPA in our modified reserpine mouse model. We found that it was effective for relieving motor symptoms of PD. The possible therapeutic efficacy of another clinical drug, pramipexole was also evaluated. Pramipexole is a dopamine non-ergoline agonist, which is used in the PD treatment (Yoo et al., 2018). Our results showed that pramipexole treatment significantly alleviated some non-motor symptoms (such as depression and pain), but not constipation and olfactory impairment, in mice. These results were in line with previous clinical observations that pramipexole treatment exerts antidepressant effects in PD patients (Dooley and Markham, 1998; Kano et al., 2008). Thus, preclinical reserpine PD models are still useful for screening new anti-PD drugs.

### 4.2 Reserpine induces alpha-synuclein deposition and autophagy impairment

The causes of alpha-synuclein deposition include gene mutations, autophagy-lysosome dysfunctions, oxidative stress in the presence of cytoplasmic dopamine, and impaired proteo-soma processing or metabolism (Ohtake et al., 2004; Wang et al., 2015; Lee et al., 2021). Inefficient clearance of alpha-synuclein can lead to cytotoxicity (Vogiatzi et al., 2008). The substantia nigra and striatum of neonatal rats treated with reserpine (5 mg/kg) on postnatal day 3 exhibit alpha-synuclein-positive inclusions (van Onselen and Downing, 2021). However, an increase in immunoreactivity to alpha-synuclein in the substantia nigra and dorsal striatum of animals treated with reserpine shows recovery after 15 days (Leão et al., 2017). In this study, we observed that reserpine treatment induced alpha-synuclein deposition both *in vitro* and *in vivo*.

Autophagy is a cellular mechanism involved in eliminating cell waste, damaged organelles, and aggregations of proteins, and it functions to maintain cellular homeostasis (Yu et al., 2020). Alpha-synuclein degradation is mediated mainly by autophagy and the lysosome (Mao et al., 2020). Autophagic flux can be evaluated by the expression of p62, LC3-II/LC3-Ⅰ and GFP-LC3 protein (Lee et al., 2015; Bai et al., 2021). In the present study, reserpine treatment upregulated the ratio of LC3-II/LC3-Ⅰ levels and that of the autophagic substrate, p62, in SH-SY5Y cells and mice brains. The p62 protein is primarily degraded during autophagy, and therefore, inhibition of lysosomal degradation of autophagic cargo causes its accumulation (Hussain et al., 2019). Thus, our data indicates that reserpine also caused autophagy impairment *in vitro* and *in vivo*.

### 4.3 The possible mechanism underlying SNCA hypo-methylation in the reserpine-induced Parkinson’s disease model

Recently, epigenetic regulation of alpha-synuclein expression has been demonstrated (McGregor et al., 2021). CpG methylation is a classical epigenetic modification that regulates gene transcription by inhibiting the binding of DNA sequences to transcription factors (Kantor et al., 2018; Unnikrishnan et al., 2019). CpG demethylation leads to transcriptional reactivation (Jones, 2012). Additionally, CpG demethylation of *SNCA* intron-1 is linked to its upregulation (Matsumoto et al., 2010). The CpG islands of *SNCA* showed demethylation, which is in line with the findings of previous studies. DNMTs, including the three active subtypes, DNMT3a, DNMT3b, and DNMT1, catalyze the transfer of methyl to cytosine; 6-OHDA and MPP^+^ induce down-regulation of DNMT3b and DNMT3a (Yang et al., 2017). Similarly, in the present study, DNMT3b was downregulated in mRNA and protein expression levels. These results suggested that CpG methylation modifications may exhibit specific patterns under different environmental conditions, which may be attributed to hypermethylation of some CpG islands and hypomethylation of others (Huidobro et al., 2013). Our findings suggested that CpG demethylation around the promoter region of *SNCA* may be regulated by DNMT3b, which was downregulated in neuronal cells and mice brains exposed to reserpine.

Elevated levels of both reactive oxygen species (ROS) and DNA methylation are observed in our results. Mounting evidence demonstrated that oxidative stress play a significant role in the development of Parkinson’s disease (Hemmati-Dinarvand et al., 2019). Interestingly, at an early stage, accompanying with the increment of alpha-synuclein, ROS accumulation was increased (Fu et al., 2018). However, the relation between ROS and DNA methylation is not well understood. ROS can influence both aspects of DNA methylation changes through different mechanisms (Wu and Ni, 2015). ROS can function as catalysts of DNA methylation (Wu and Ni, 2015). In our study, we found that ROS and SNCA hypo-methylation were induced by reserpine. However, the cause and effects are elusive. Maybe, it is possible that ROS production is an important contributor to SNCA hypo-methylation and synuclein accumulation. However, how ROS regulates DNA methylation changes warrants further investigation.

However, there are several possible drawbacks to our study. First, the incidence of Parkinson’s disease is higher in men than in women, thus studies taking into account this sexual bias were warranted. Second, the recovery time is unclear for alpha-synuclein and TH expression. Third, the mice model was established by oral administration, and the related indicators for the brain-gut axis were not evaluated. Follow up research to address these issues is thus required.

In conclusion, we demonstrated that reserpine induces PD-like motor and non-motor symptoms in mice and flies. Epigenetic upregulation of alpha-synuclein and autophagy impairment are involved in the pathogenesis of reserpine-induced PD. through Herein we highlight some of the neurochemical changes induced by reserpine, such as alpha-synuclein deposition and reduction of TH in the substantia nigra. According to these findings, we proposed that the reserpine models may be used as a valuable tool in preclinical studies on motor and non-motor symptoms of PD.

## Data Availability

The original contributions presented in the study are included in the article/Supplementary Material; further inquiries can be directed to the corresponding authors.
